# Phosphorylation of CrkL S114 induced by common gamma chain cytokines and T-cell receptor signal transduction

**DOI:** 10.1038/s41598-021-96428-y

**Published:** 2021-08-20

**Authors:** Armando Estrada, Alejandro C. Rodriguez, Georgialina Rodriguez, Alice H. Grant, Yoshira M. Ayala-Marin, Amy J. Arrieta, Robert A. Kirken

**Affiliations:** 1 Department of Biological Sciences, The University of Texas At El Paso, El Paso, TX 79968 USA; 2 Border Biomedical Research Center, The University of Texas At El Paso, El Paso, TX 79968 USA

**Keywords:** Cell biology, Immunology, Molecular biology

## Abstract

T-cell activation and cellular expansion by common gamma chain cytokines such as Interleukin-2 is necessary for adaptive immunity. However, when unregulated these same pathways promote pathologies ranging from autoimmune disorders to cancer. While the functional role of Interleukin-2 and downstream effector molecules is relatively clear, the repertoire of phosphoregulatory proteins downstream of this pathway is incomplete. To identify phosphoproteins downstream of common gamma chain receptor, YT cells were radiolabeled with [^32^P]-orthophosphate and stimulated with Interleukin-2. Subsequently, tyrosine phosphorylated proteins were immunopurified and subjected to tandem mass spectrometry—leading to the identification of CrkL. Phosphoamino acid analysis revealed concurrent serine phosphorylation of CrkL and was later identified as S114 by mass spectrometry analysis. S114 was inducible through stimulation with Interleukin-2 or T-cell receptor stimulation. Polyclonal antibodies were generated against CrkL phospho-S114, and used to show its inducibility by multiple stimuli. These findings confirm CrkL as an Interleukin-2 responsive protein that becomes phosphorylated at S114 by a kinase/s downstream of PI3K and MEK/ERK signaling.

## Introduction

Dysfunction in cell signaling can lead to the manifestation of immunological disorders such as immunodeficiency, autoimmunity and hematopoietic malignancies^1–3^. Many of these conditions are the result of abnormal regulation of the common gamma chain (γ_c_) family of cytokines^2,4–6^ involved in T-cell and natural killer (NK) cell function. This family consists of Interleukins (IL) IL-2, IL-4, IL-7, IL-9, IL-15, and IL-21 which mediate T-helper development, T-cell proliferation, and homeostasis^4^. In addition, IL-2 drives effector T-cell clonal expansion maintains peripheral T-cell tolerance, regulates immune homeostasis through T regulatory cell survival and supports activation induced cell death (AICD) of T-cells^4,7,8^.

γ_c_ cytokines exert biological effects through their recruitment and activation of Janus Tyrosine Kinases (JAKs), Signal Transducers and Activators of Transcription (STAT), Phosphoinositide 3-Kinase (PI3K) and the MAP Kinase (MAPK) pathways^2,9^. Many of these pathways are also required for signal transduction by the T-cell receptor (TCR) and the co-stimulatory molecule, CD28^9^. In response to foreign antigens TCR aggregation results in tyrosine kinase recruitment and subsequent phosphorylation of cytoplasmic receptor residues triggering the PKA, PI3K and MAPK/ERK pathways^9–13^. The overlap between IL-2 and TCR signaling is not surprising given that TCR/CD28 activity leads to the expression of the IL-2α receptor necessary for full T-cell activation^10^. Additionally, a strong correlation exists between antigen load and IL-2^14^. T-cell dynamics have been linked to integrative signals by downstream Extracellular Signal-Regulated Kinase (ERK) induced by MEK^15^. While the functional role of IL-2 and downstream effector molecules is relatively clear, promoting differentiation, proliferation, and survival of T cells, the various co-regulatory pathways involved in this cascade are much less understood.

To better understand this signaling network, we sought to identify novel IL-2 phospho-Tyrosine (pY) inducible proteins. NK-like YT cells were metabolically labeled with [^32^P]-orthophosphate and stimulated with IL-2. The resulting IL-2 induced tyrosine phosphorylated proteins were assessed by mass spectrometry and phosphoamino acid analysis resulted in the detection of a 37 kDa protein identified as -Crk-like protein (CrkL). A ubiquitously expressed member of the Crk family of adaptor proteins^16^, CrkL possesses a single amino-terminal Src-homology-2 (SH2) domain followed by two Src-homology-3 (SH3) domains with no known enzymatic activity. The two SH3 domains are commonly referred to as amino-terminal SH3 (nSH3) and carboxyl-terminal SH3 (cSH3)^28,29^. CrkL traditionally serves as a signaling adaptor by linking activated membrane receptors to downstream signaling effectors, through the interaction of their SH3 and SH2 domains^17,18^. A regulatory phosphosite, Y207, within CrkL has been shown to inhibit its adaptor function by causing a conformational change where pY207 interacts directly with its own SH2 domain^19,20^. CrkL tyrosine phosphorylation has been identified in response to IL-2^21^ yet the precise roles of this adaptor protein within this pathway remains elusive. Less is known about serine/threonine phosphoregulatory sites within CrkL downstream of IL-2. Here, a novel phospho-site, S114, within CrkL was identified downstream of IL-2, TCR and other effector pathways. Notably, phosphorylation of CrkL S114 was dependent on serine/threonine kinases downstream of PI3K and MAPK/ERK pathways that are induced by IL-2.

## Results

### CrkL is tyrosine and serine phosphorylated in response to IL-2

**Figure 1:**
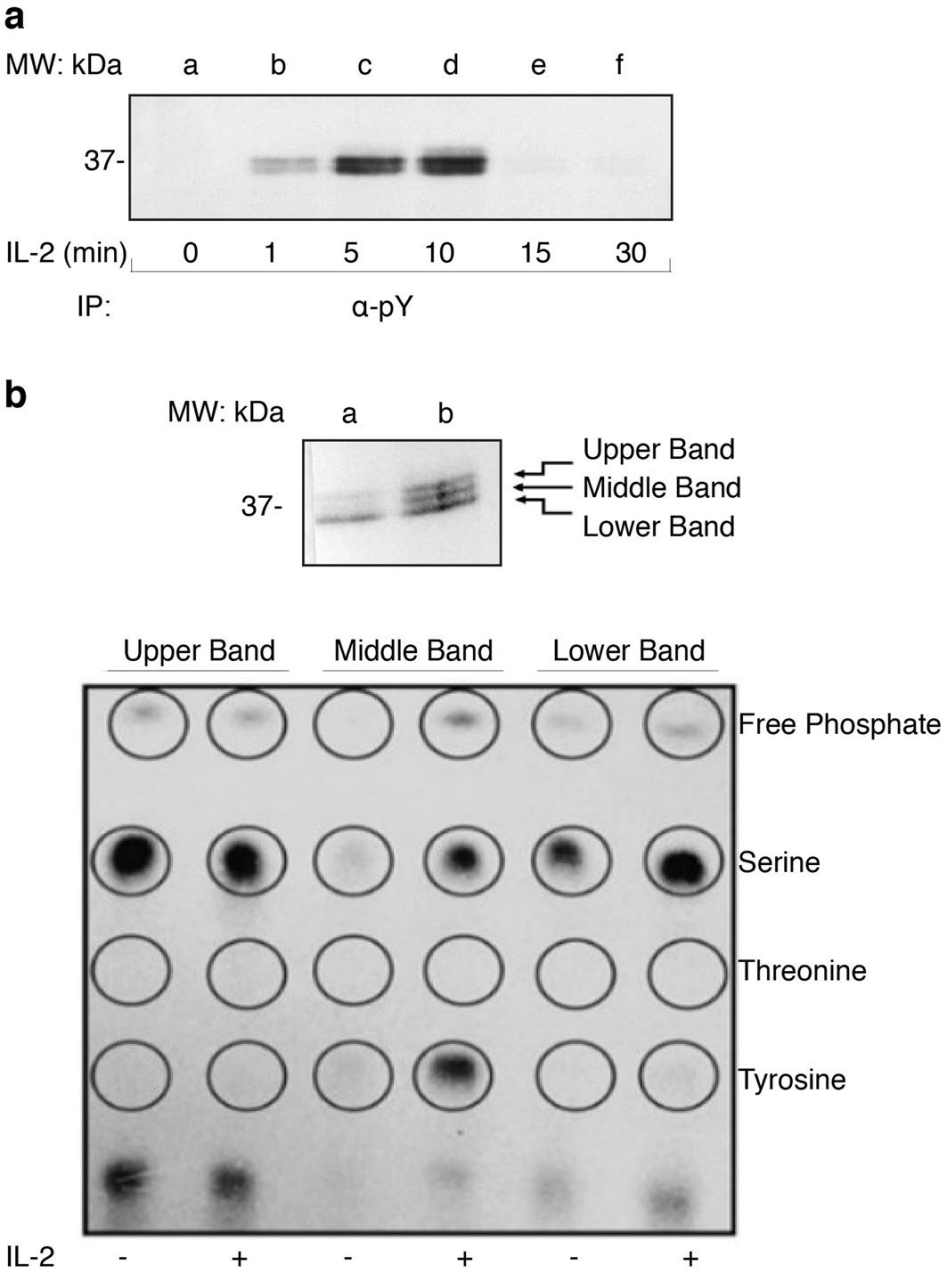
CrkL is tyrosine and serine phosphorylated in response to IL-2. (**a**) YT cells were grown to confluency and either left untreated (lane **a**) or stimulated with IL-2 for 1, 5, 10, 15, and 30 min (lanes **b** –**f**). Cellular proteins were subjected to immunoprecipitation with anti-phosphotyrosine agarose conjugated antibodies (α-pY Agarose) and Western blotted for α-phosphotyrosine (α-pY). Full-length blots are displayed in Supplementary Fig. S1a. (**b**) YT cells were stimulated (−/ +) with IL-2 for 10 min (lanes **a** and **b** respectively) and cellular lysate was Western blotted with α-CrkL (top panel). Full-length blots are displayed in Supplementary Fig. S1b. Multiple bands were excised and subjected to limited hydrolysis before being spotted on a thin layer cellulose-acetate gel and separation by electrophoresis (bottom panel). Migration of standards was visualized with ninhydrin and radiolabeled samples detected by autoradiography.

As discussed previously, IL-2 triggers multiple signaling pathways that mediate both NK cell and T-cell function. Here, the human NK cell line, YT, was used to identify novel IL-2 inducible phospho-proteins, specifically tyrosine phosphorylated proteins as they are widely accepted to be activated signaling molecules within the JAK/STAT pathway. Cells were stimulated with IL-2 from 0 to 30 min and cell lysates were immunoprecipitated with α-pY conjugated beads in combination with phosphoamino acid analysis as previously described^22^. Multiple IL-2 inducible phospho-proteins, including a band at 37 kDa were detected (Fig. 1a). This band showed robust and short-lived tyrosine phosphorylation lasting between 5 and 10 min that reverted to basal levels after 15 min post IL-2 stimulation, suggesting regulation for transient activity. Next, YT cells were stimulated with IL-2 for 10 min, immunoprecipitated with α-pY agarose and subjected to SDS-PAGE where the 37 kDa band was excised and confirmed as CrkL through mass spectrometry. To identify CrkL phosphorylation patterns of tyrosine, serine and threonine, YT cells were metabolically labeled with [^32^P]-orthophosphate, stimulated with or without IL-2, immunoprecipitated with CrkL antibodies, separated by SDS-PAGE and subjected to autoradiography. Each band (upper, middle and lower) was excised and subjected to phosphoamino acid analysis (Fig. 1b, top panel). The upper and lower bands displayed constitutive serine phosphorylation; however serine phosphorylation was slightly lighter in the lower band of the non-stimulated lane compared to the IL-2 stimulated lane, indicating some level of inducibility. The middle band showed inducible tyrosine and serine, but not threonine, phosphorylation (Fig. 1b, bottom panel). These results suggest CrkL is an IL-2 inducible protein that leads to the rapid induction of tyrosine and serine, but not threonine phosphorylation.

#### IL-2 and TCR stimulation induce post-translational modifications of CrkL detected by mobility shift

**Figure 2:**
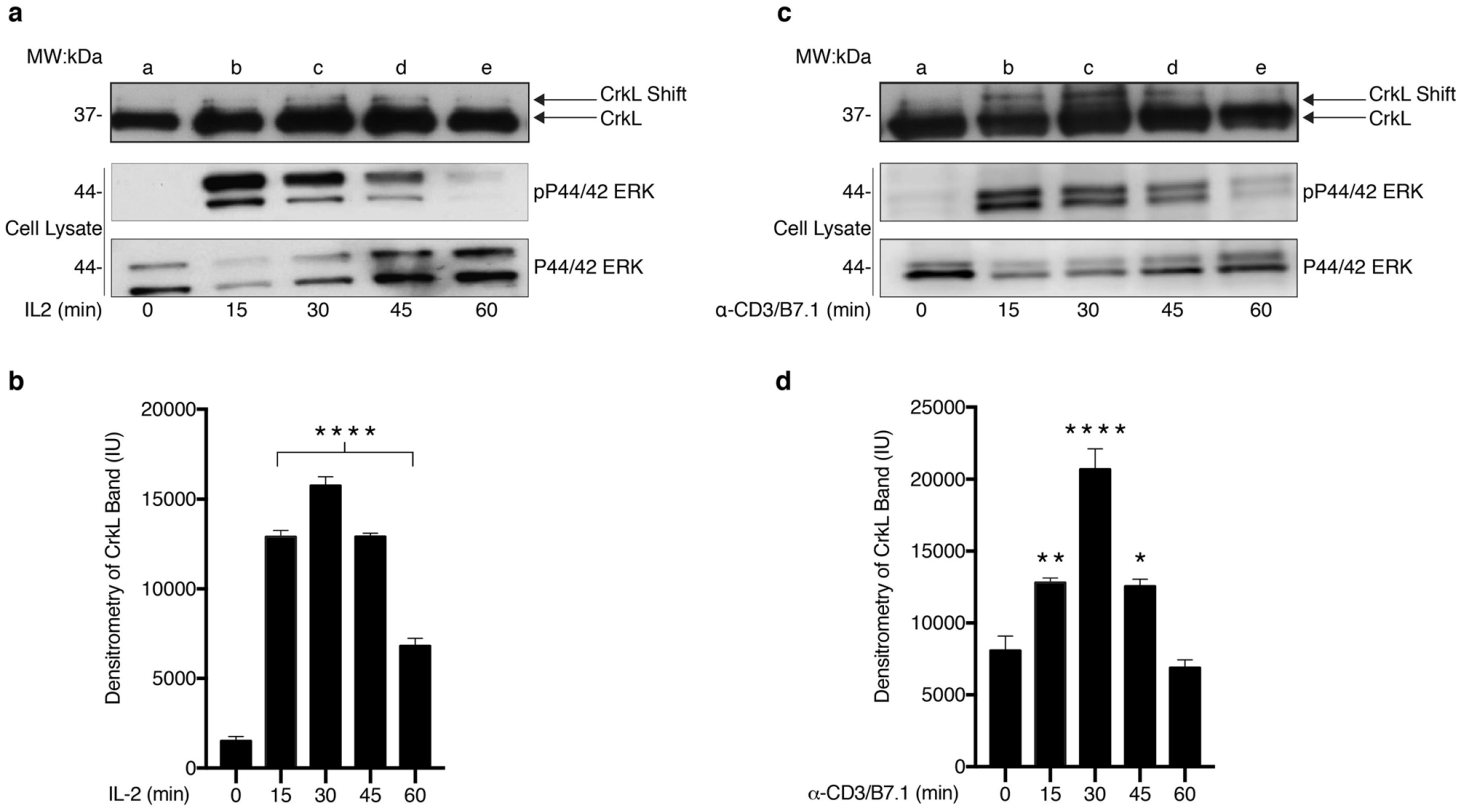
IL-2 and TCR stimulation induce post-translational modifications of CrkL detected by mobility shift. (**a**) Quiescent Kit225 cells were either left untreated (lane **a**) or stimulated with 100 nM IL-2 for 0, 15, 30, 45, or 60 min (lanes **b**–**e**). Cells were then subjected to immunoprecipitation for CrkL and protein was Western blotted with α-CrkL antibodies. Total cell lysate input was reserved and Western blotted for pP44/42 (α-pERK) (middle panel) and total P44/42 (α-ERK) (bottom panel). Full-length blots are displayed in Supplementary Fig. S2ab. (**b**) Densitometry analysis was performed on CrkL mobility shift from three independent experiments (n = 3) and the mean Intensity Units (IU) for each condition depicted in the bar graph. (**c**) Jurkat cells were either left untreated (lane **a**) or stimulated with α-CD3 (5 µg) monoclonal antibodies and rhB7.1 (100 ng) for 0, 15, 30, 45, or 60 min (lanes **b**–**e**). Cell lysate was subjected to immunoprecipitation for CrkL, protein and Western blotted with α-CrkL antibodies. Total cell lysate input was reserved and Western blotted for α-pERk (middle panel) and total ERK (bottom panel). Full-length blots are displayed in Supplementary Fig. S2cd. (**d**) Densitometry analysis was performed on CrkL mobility shift from three independent experiments (n = 3) and the mean Intensity Units (IU) for each condition depicted in the bar graph. (****) denotes *P* > 0.0001, (**) denotes *P* > 0.003, (*), denotes *P* > 0.05.

Our previous findings revealed that IL-2, a critical driver of NK and T-cell expansion, induced CrkL phosphorylation. To investigate the dynamics of CrkL phosphorylation within the IL-2 signaling pathway we utilized T cells (Kit225) that depend on this pathway for survival^23,24^. Quiescent Kit225 cells were stimulated with IL-2 (0–60 min), cell lysates were subjected to CrkL immunoprecipitation, separated by SDS-PAGE and Western blotted against CrkL that was analyzed for mobility shift, indicative of changes in phosphorylation. This interpretation is supported by the detection of changes in tyrosine, and serine phosphorylation in response to IL-2 in the shifted CrkL band from the previous phosphoamino acid analysis. Altered gel mobility of CrkL peaked at 30 min (Fig. 2a, top panel), and receded 1-h post-stimulation while displaying similar kinetics to ERK activation (Fig. 2a, middle panel). Densitometric analysis of the upper CrkL band demonstrated reproducible results in three independent trials (Fig. 2b)**.** The T-cell leukemia cell line, Jurkat, revealed parallel findings in CrkL mobility with mimicked antigenic stimulation (TCR stimulation) by anti-CD3 monoclonal antibodies (α-CD3), in combination with recombinant human B7.1 (rhB7.1). Specifically, CrkL mobility shift, mirrored ERK activation (Fig. 2c). Densitometric analysis of the upper CrkL band (three independent experiments) revealed that TCR activation induced similar CrkL activation kinetics (Fig. 2d) as those observed in cells stimulated with IL-2 (see Fig. 2a, b); with the upper CrkL band peaking at 30 min, followed by a decline of signal at 45 min and loss of signal after 60 min.

#### Common gamma chain cytokines induce post-translational modifications of CrkL detected by mobility shift

**Figure 3:**
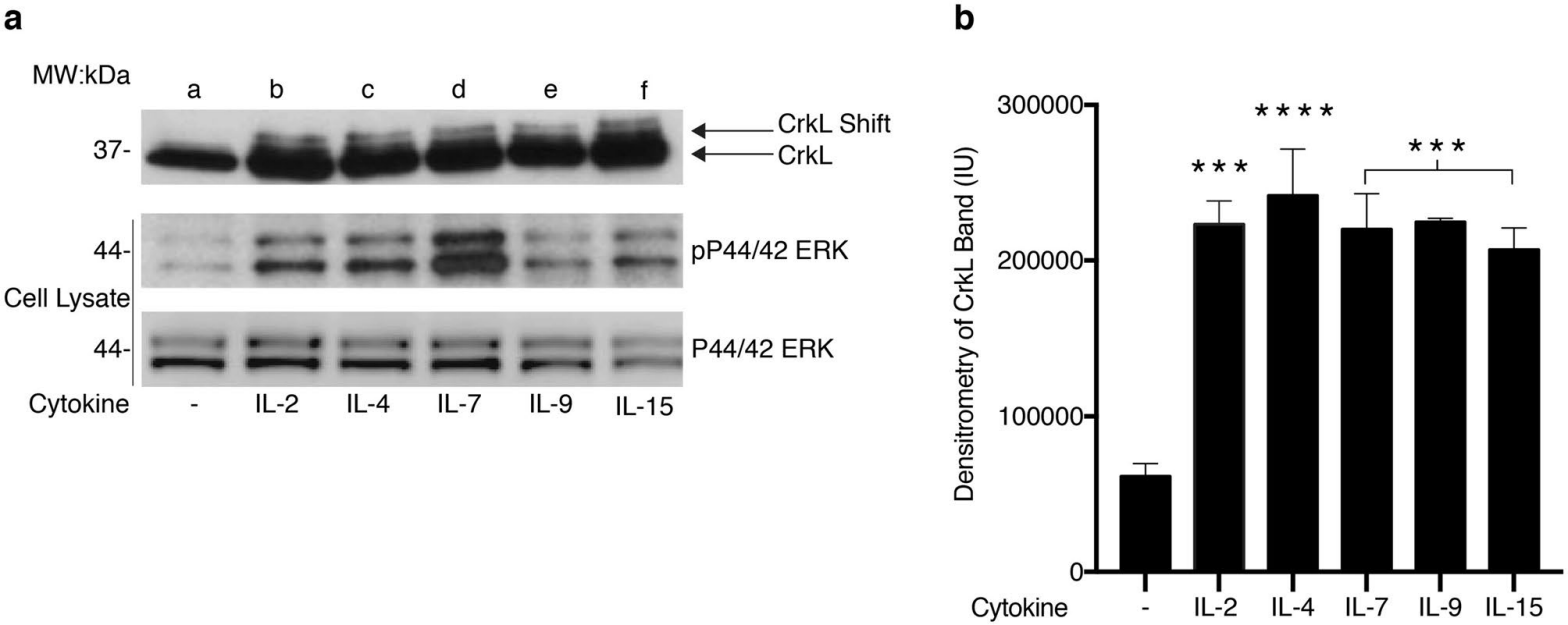
Common gamma chain cytokines induce post-translational modifications of CrkL detected by mobility shift. (**a**) Quiescent Kit225 cells were left untreated (lane **a**) or stimulated as follows: 100 nM IL-2 (lane **b**), 100 nM IL-4 (lane **c**), 100 nM IL-7 (lane **d**), 100 nM IL-9 (lane **e**) or 100 nM IL-15 (lane **f**) for 30 min. Cells were then subjected to immunoprecipitation with α-CrkL antibodies, separated by SDS-PAGE and Western blotted with α-CrkL. Total cell lysate input was reserved, separated by SDS-PAGE and Western blotted for α-pERK (middle panel) and total α-ERK (bottom panel). Full-length blots are displayed in Supplementary Fig. S3a. (**b**) Densitometry analysis was performed on three independent experiments (n = 3) and the mean Intensity Unit (IU) for each condition represented in the bar graph. (****) denotes *P* > 0.0001, (***) denotes *P* > 0.001.

T-cells respond to multiple extracellular stimuli, including cytokines belonging to the γ_c_ family. To determine whether these stimuli also alter CrkL mobility, quiescent Kit225 cells were stimulated with either IL-2, IL-4, IL-7, IL-9 or IL-15 for 30 min at 37 °C. Each cytokine assessed induced higher gel mobility of CrkL (Fig. 3a), which was analogous to the activation profile observed with phosphorylated ERK (Fig. 3a, middle panel). Densitometric analysis of the upper CrkL band revealed similar results in three independent experiments (Fig. 3b). Cumulatively, these data suggest that CrkL could play a role in regulating T-cell proliferation (IL-2), development (IL-4, IL-7, and IL-15), differentiation (IL-2, IL-4, IL-7 and IL-15) and survival (IL-2, IL-7).

### Identification of CrkL S114 as an IL-2 and TCR inducible phospho-site

**Figure 4:**
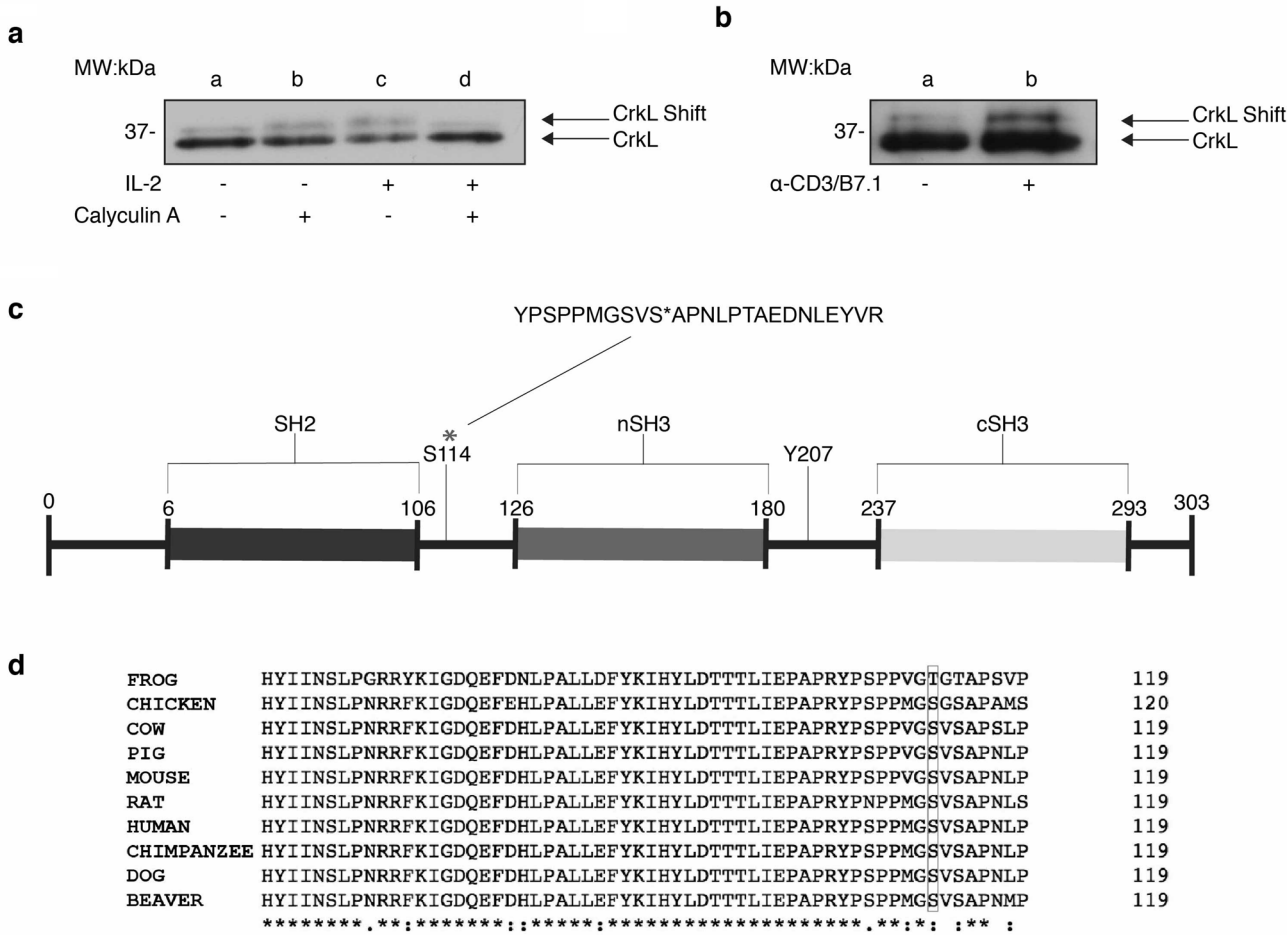
Identification of CrkL S114 as an IL-2 and TCR inducible phospho-site. (**a**) Quiescent Kit225 cells were either untreated (lane **a**) or treated with 100 nM calyculin A (lane **b**), 100 nM IL-2 (lane **c**) or 100 nM calyculin A prior to 100 nM IL-2 stimulation (lane **d**) for 30 min. Cell lysates were immunoprecipitated with α-CrkL antibodies and Western blotted for CrkL. Duplicate samples were immunoprecipitated, separated, Coomassie-Blue stained and bands excised for Tandem Mass Spectrometry analysis. (**b**) Jurkat cells were either untreated (lane **a**) or stimulated with 5 µg of α-CD3 monoclonal antibodies and 100 ng of rhB7.1 (lane **b**) for 15 min. Cell lysate was immunoprecipitated with CrkL antibodies and Western blotted for CrkL. Full-length blots are displayed in Supplementary Fig. S4ab. Duplicate samples were immunoprecipitated, separated, Coomassie-Blue stained and bands excised for Tandem Mass Spectrometry analysis. (**c**) Schematic diagram of human CrkL depicting the location of the newly identified phospho-site S114, LC–MS/MS peptide fragment containing S114 (above), and the previously identified Y207 site. (**d**) CrkL protein sequence alignment from various species.

Given CrkL’s mobility shift in response to IL-2, TCR and the γ_c_-cytokines we utilized mass spectrometry to identify residues accountable for CrkL shifts indicative of post-translational modifications. This analysis would be complementary to the general tyrosine and serine phosphorylation observed in CrkL (see Fig. 1) by identifying specific phosphorylation sites within distinct CrkL bands. To produce the observed shifts induced by IL-2 or TCR stimulation (see Fig. 2.) Kit225 cells were stimulated with either IL-2, calyculin A (CA) or a combination of both; Jurkat cells were stimulated with anti-CD3 and rhB7.1 to mimic TCR stimulation. Cell lysates were immunoprecipitated using CrkL antibodies and separated by SDS-PAGE, followed by Coomassie-blue staining to visualize protein bands. Detectable bands (37 kDa) from unstimulated and stimulated cells were then excised and sent for mass spectrometry analysis. Concurrently processed samples were separated by SDS-PAGE and Western blotted for CrkL to confirm altered protein gel mobility in response to IL-2 (Fig. 4a) and TCR stimulation (Fig. 4b). Phosphorylation sites were compared between unstimulated and stimulated cells to identify inducible phosphorylation events. This strategy identified S114, a previously unreported CrkL phospho-residue, that is induced by both IL-2 and TCR stimulation Supplementary Fig. S4 (Fig. 4a, lane c, lower band; Fig. 4b, lane b, upper band, respectively). XCorr and Ascore values for S114 phospho-peptides are included in Supplementary Table 1. Serine phosphorylation, including S114, was detected within the lower band of IL-2 stimulated Kit225 cells, and indeed we observed a collapse of the CrkL shift in response to calyculin A pre-treatment. The detected peptide fragment containing phosphorylated S114 is positionally located in a linker region between the SH2 and the nSH3 domains within CrkL (Fig. 4c). A protein sequence alignment shows CrkL S114 conservation among mammals, and minimally preserved among diverse species including Western-clawed frog (Xenopus Tropicalis) and chicken (Gallus gallus) (Fig. 4d) (Uniprot BLAST). Additionally, the identified region surrounding S114 is unique to CrkL amongst the Crk family of proteins.

#### The CrkL pS114 antibody preferentially recognizes phosphorylated peptides and detects CrkL S114 phosphorylation induced by several cytokines

**Figure 5:**
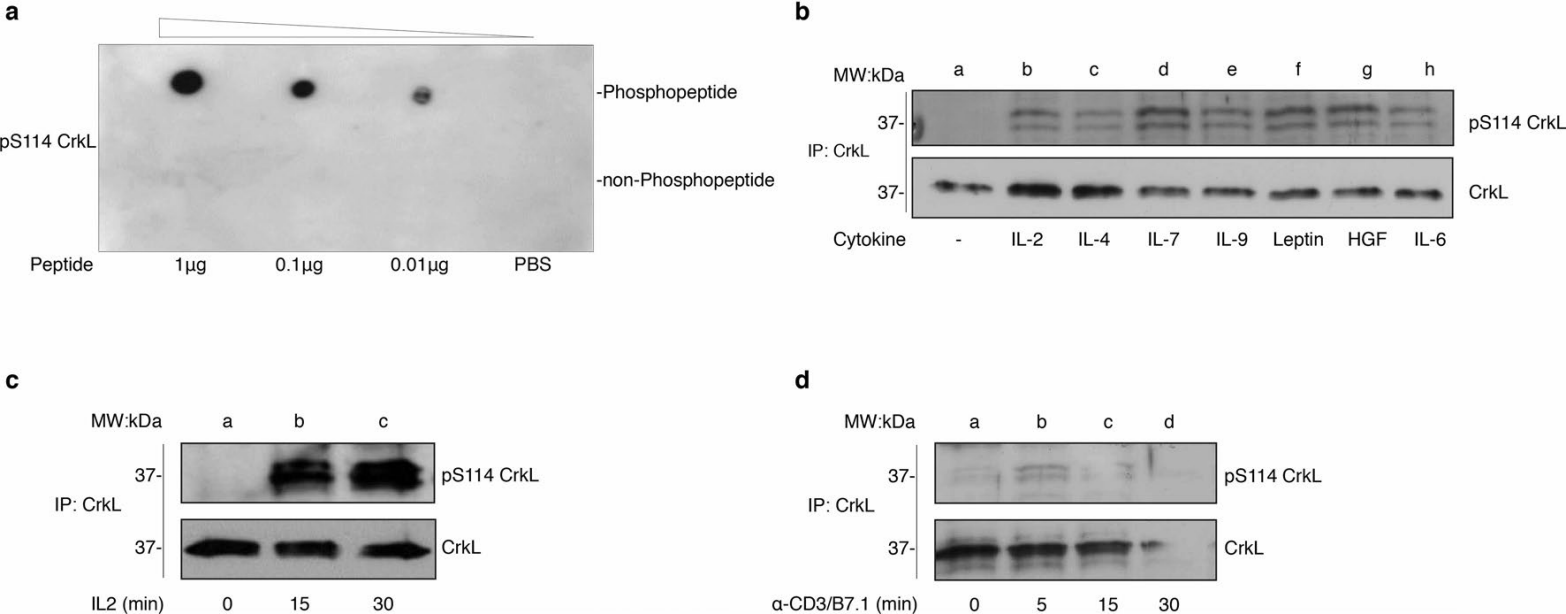
The CrkL pS114 antibody preferentially recognizes phosphorylated peptides and detects CrkL S114 phosphorylation induced by several cytokines. (**a**) Phosphopeptide (MGSV^33^APNLPTAEDC) and non-phosphopeptide (MGSVSAPNLPTAEDC) were spotted onto PVDF membrane in decreasing amounts along with vehicle control (PBS). The resulting membrane was Western blotted using α-pS114 polyclonal antibodies. (**b**) Quiescent Kit225 cells were stimulated with various growth factors cytokines including IL-2 (100 nM, lane **b**), IL-4 (100 nM, lane **c**), IL-7 (100 nM, lane **d**), IL-9 (100 nM, lane **e**), Leptin (10 ng/ml, lane **f**), HGF (100 ng/ml, lane **g**) or IL-6 (400 ng/ml, lane **h**) for 15 min. Cell lysate was immunoprecipitated for CrkL, Western blotted using α-pS114 polyclonal antibodies and re-blotted for total CrkL. Shown is representative data of two independent experiments (n = 2). Full-length blots are displayed in Supplementary Fig. S5a. (**c**) Quiescent Kit225 cells were stimulated without (lane **a**), with 100 nM IL-2 for 15 min (lane **b**) or 30 min (lane **c**). Cell lysates were immunoprecipitated using CrkL antibodies, Western blotted using α-pS114 polyclonal antibodies and re-blotted for total CrkL. Full-length blots are displayed in Supplementary Fig. S5b. (**d**) Jurkat cells were stimulated with α-CD3 (5 µg) monoclonal antibodies and rhB7.1 (100 ng) for 0, 5, 15, and 30 min (lanes **a**–**d**). Cell lysate was subjected to immunoprecipitation for CrkL, protein and Western blotted with polyclonal α-pS114 CrkL antibodies. Full-length blots are displayed in Supplementary Fig. S5c.

To elucidate the regulation of serine phosphorylation within CrkL, a novel phospho-S114 (pS114 CrkL) polyclonal antibody was generated against this site. Antibodies were tested by dot blot analysis for specificity and selectivity to recognize phosphopeptide vs. non-phosphopeptide (Fig. 5a). Next, quiescent Kit225 cells were challenged with a panel of growth factor cytokines to establish whether they could induce CrkL S114 phosphorylation. This panel included the γ_c_ family of cytokines; leptin, involved in T-cell metabolism; Hepatocyte Growth Factor (HGF) involved in T-cell motility and IL-6 involved in T-cell survival^25–27^. CrkL S114 phosphorylation was observed in response to the latter cytokines at varying degrees (Fig. 5b). Kit225 cells stimulated with IL-2 induced marked activation of pS114 CrkL detected most intensely at 15 and 30 min (Fig. 5c). Jurkat cells stimulated with anti-CD3 monoclonal antibodies and rhB7.1 for 5, 15 or 30 min (Fig. 5d) displayed a modest yet distinct pattern peaking at 5 min. These data suggest that phosphorylation of CrkL S114 is regulated by several cytokines and perhaps a convergence point for multiple T-cell stimuli.

#### CrkL S114 phosphorylation is dependent on IL-2-induced activation of mTOR and MEK/ERK pathways

**Figure 6:**
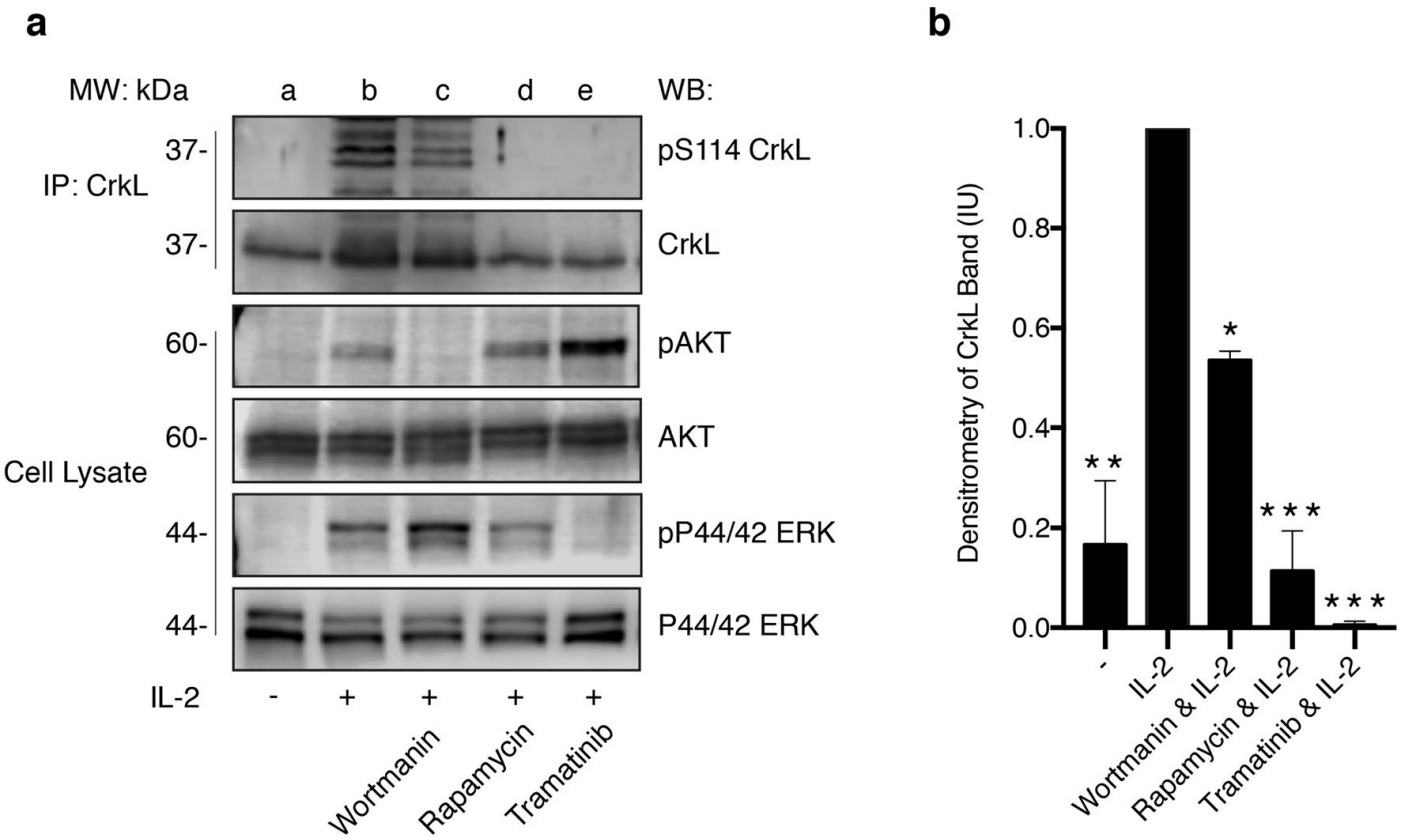
CrkL S114 phosphorylation is dependent on IL-2-induced activation of mTOR and MEK/ERK pathways. (**a**) Quiescent Kit225 cells were either untreated (lane **a**), stimulated with IL-2 only (lane **b**), or pre-treated with 50 μM wortmannin (lane **c**), 100 nM rapamycin (lane **d**), and 100 nM tramatinib (lane **e**) for 1 h prior to IL-2 stimulation for 30 min. Cell lysate was immunoprecipitated for CrkL protein, Western blotted using α-pS114 and re-blotted for total CrkL. Total cell lysate reserved was Western blotted using α-pERK and α-pAKT, and then reblotted for α-ERK and α-AKT. Full-length blots are displayed in Supplementary Fig. S6a. (**b**) Densitometry analysis was performed on CrkL pS114 from two independent experiments (n = 2) and the mean Intensity Unit (IU) for each condition represented in the bar graph. (***) denotes *P* > 0.001, (**) denotes *P* > 0.01, (*) denotes *P* > 0.05.

To identify the serine/threonine kinases which phosphorylate CrkL S114, quiescent Kit225 cells were pre-treated with kinase inhibitors wortmannin, rapamycin, and trametinib—which are known to be potent inhibitors of PI3K, mTOR and MEK, respectively. Inhibition of mTOR and MEK activation prevented S114 phosphorylation in response to IL-2 treatment (Fig. 6, lane d and e). In contrast, inhibition of PI3K was less effective in the inhibition of CrkL S114 phosphorylation (Fig. 6, lane c). The specificity and selectivity of each individual kinase inhibitor was analyzed using Western blot for pAKT/AKT (wortmannin, PI3K inhibitor, lane c) and pERK/ERK (trametinib, MEK inhibitor, lane e) (Fig. 6).

## Discussion

T-cell functions are largely regulated through two fundamental signaling networks: the TCR and γ_c_-cytokine mediated signaling pathways^28^. The TCR is critical for T-cell development, homeostasis, and activation. Meanwhile γ_c_-cytokine signaling, is fundamental to cell proliferation, subset specialization, maintenance and memory generation^29,30^. The γ_c_ family of cytokines (IL-2, IL-4, IL-7, IL-9, IL-15 and IL-21), signaling through the JAK/STAT pathway, promote homeostasis and elicit an immune response following TCR engagement^4^. It is important, therefore, to identify shared effector proteins that may facilitate crosstalk between these two signaling pathways. Here, CrkL activation was seen downstream many of the latter pathways. Specifically, IL-2 induced CrkL serine and tyrosine, but not threonine, phosphorylation, see Fig. 1. Furthermore, TCR activation and γ_c_-cytokines induced CrkL mobility shifts indicative of changes in phosphorylation, see Figs. 2 and 3. Perhaps the CrkL adaptor protein facilitates signal transduction of converging immune cascades.

The Crk family, have been identified to interact with over 40 cellular proteins^26^. Notably, CrkL has been shown to play a critical role in TCR complex formation with CasL tyrosine kinase by recognizing phosphorylated tyrosine through its SH2 domain^31^. Other groups have shown CrkL to be important in T-cell activation through its promotion of C3G and RAP guanine exchange factor-1 (RAP1) complex formation, which enhances guanine tri-phosphate’s (GTP) binding and subsequent MAPK activation^17–19^. This latter pathway is also downstream of IL-2 and perhaps facilitated by CrkL. Consistent with its role in TCR co-stimulation, studies have shown that CrkL plays a role in altering T-cell cytokine secretion^31^. To date, CrkL has been linked to a number of physiological processes that can have aberrant consequences if unregulated, such as cell migration, cell cycle progression, and apoptosis^20^. Henceforth, multiple studies suggest CrkL is an important factor in the study of cancer. Clinical observations suggest increased CrkL expression is directly correlated with aggressive and invasive cancer phenotypes in ovarian and lung cancer^7^. CrkL has furthermore been reported to have transformative properties, as overexpression in mammalian cells has resulted in cell transformation^7^. In fact, the phosphorylation of CrkL at Y207 is considered an indicator of cancer remission in Chronic Myeloid Leukemia patients after imatinib treatment^12^, and previously was the only identified phospho-site known to regulate CrkL activity.

In this study, a novel phosphorylation site was identified within CrkL located between SH2 and nSH3, at residue S114, see Fig. 4. Perhaps, phosphorylation of the S114 site facilitates CrkL adaptor function unlike pY207. Interestingly a quick database search of the Catalogue of Somatic Mutations in Cancer revealed a missense CrkL mutation, S114L (Genetic Mutation ID: COSV62883704)^32^, which has been deemed pathogenic^33^. Consistent with the findings reported here, a missense mutation at S114 could potentially lead to detrimental effects on multiple signaling pathways including γ_c_-cytokines and T-cell activation. Inducibility of CrkL pS114 by multiple stimuli exemplifies that CrkL may act as a convergence point for many co-signaling pathways associated with T-cell function. The pharmacological inhibitor studies utilizing serine/threonine kinase inhibitors point to regulation of CrkL S114 by multiple signaling cascades, as phosphorylation was blocked by inhibitors of mTOR (Rapamycin) and MEK/ERK [Trametinib (see Fig. 6)]. This is not surprising given that an abundance of serine/threonine kinases have been identified in regulating γ_c_ and TCR signaling pathways^34^.

One possible scenario in which CrkL S114 becomes phosphorylated is to integrate signals involving cross-talk between the three signaling events required for full T-cell activation^10^. In signal 1, the antigen is presented by MHC class I or II receptor engagement with the TCR, which in turn activates multiple signaling kinase cascades such as LCK, Zap70, ERK and PKA pathways^10^. Signal 2 is initiated by the activation of CD28 engagement with the B7.1 molecule, which activates downstream signaling pathways such as PI3K/AKT and JNK. This activation of signal 2 leads to the expression of the γ_c_-cytokine receptors such as IL-2^10^. These γ_c_-cytokine receptors induce the final signal (signal 3) that is responsible for full T-cell activation and proliferation, which promotes the activation of several signaling pathways such as JAK/STAT, ERK, and mTOR pathways. CrkL S114 can become phosphorylated in response to TCR activation of ERK and mTOR. “Activated” CrkL can then associate with molecules from signal 3 to transduce proliferative and survival signals. It is therefore likely that activity from signal 1 promotes adaptor molecule activation for signal 3 through phosphorylation of CrkL to promote full activation. IL-2 induced ERK activation has long been considered critical to the proliferation of T-cells^35^. As shown here, CrkL S114 phosphorylation can also be spurred by IL-2 activation, which may be facilitated by a positive feedforward loop that is important in sustaining the necessary activation signal needed to avoid AICD or various T-cell mediated functions. Future studies are needed to validate the role of CrkL in crosstalk between TCR and γ_c_ pathways. However, it is clear CrkL phospho regulation occurs downstream of multiple T-cell stimuli. Given the promiscuity of the CrkL adapter protein it may serve as a potential target for T-cell mediated immune disorders or cancers.

## Methods

### Cell culture and treatment

Human cell lines used in this study included YT, NK-like cells derived from a NK-Non-Hodgkin’s lymphoma, Kit225, T lymphocyte derived from a Chronic Lymphocytic Leukemia, and Jurkat. T lymphoblast derived from a Acute T cell Leukemia. The Kit225 cell line was kindly provided Dr. J. Johnston, Queens University, UK^23^ while all other cell lines were purchased from ATCC (http://www.atcc.org/)^36^, having met all consent policies and standards required for procurement of human biospecimens. All experimental protocols utilizing human-derived cell lines were approved by The University of Texas at El Paso in accordance with Human Research Oversight and Compliance and Institutional Review Board committee guidelines and regulations. Similarly, all methods were carried out in accordance with guidelines and regulations from the latter institutional committees. Cell lines were maintained in RPMI-1640 supplemented with 10% fetal bovine serum (FBS, Atlanta Biologicals), 2 mM L-Glutamine and 50 mg/ml penicillin–streptomycin. Kit225 cells were supplemented with 10 IU recombinant human IL-2 (rhIL-2; NCI Preclinical Repository). Kit225 cells were made quiescent by CO_2_ stripping for three short intervals (20 s) to acidify the media to liberate IL-2, followed by centrifugation, cell pellet washes with RPMI-1640 (FBS free) and overnight incubation in FBS free RPMI-1640. Quiescent cells were stimulated with rhIL-2 (100 nM), rhIL-4 (100 nM), rhIL-7 (100 nM), rhIL-9 (100 nM), rhIL-15 (100 nM) (NCI Preclinical Repository), Leptin (62.5 nM, Santa Cruz), Hepatocyte growth factor (HGF 2.5 ng/ml) or rhIL-6 (2 ng/ml, Peprotech) at 37 °C for the times indicated. Calyculin A (100 nM, PP2A inhibitor, Invitrogen) treatment was performed for 20 min at 37 °C prior to cytokine stimulation. Similarly, Jurkats were made quiescent by CO_2_ stripping (as described) and then stimulated with α-CD3 (5 µg) monoclonal antibodies and rhB7.1 (100 ng) at 37 °C for the times indicated. Inhibitor studies using Kit225 cells included: wortmannin (50 μM, PI3K inhibitor, Millipore), rapamycin (100 nM, mTOR inhibitor, Millipore), and trametinib (100 nM, MEK inhibitor, Selleck Chemicals), performed for 1 h at 37 °C. Subsequent IL-2 treatment was performed for 30 min at 37 °C.

### Antibodies, solubilization of proteins, immunoprecipitation, western blot, dot blot and mass spectrometry analysis

Cells were pelleted, lysed and subjected to immunoprecipitation, then analyzed by Western blot as previously reported^37^. Total cell lysate (TCL) protein concentration was quantified using the bicinchoninic acid method (Pierce), with 40 µg of total protein loaded per lane and separated by SDS-PAGE. Immunoprecipitations were performed as previously described^37^ using anti-CrkL antibodies (Santa Cruz, C-20) or anti-phosphotyrosine (α-pY) conjugated agarose (Millipore, 4G10). The following antibodies were used for Western blot analysis, according to manufacturer instructions: anti-pERK (Cell Signaling), anti-ERK (Cell Signaling), anti-pY (Millipore, 4G10), anti-AKT (Cell Signaling), anti-pAKT (Cell Signaling), and anti-CrkL (Santa Cruz, B-1). Western blot assays were developed using horseradish peroxidase-conjugated secondary IgG antibodies (Kirkegaard and Perry Laboratories) and visualized using enhanced chemiluminescence and X-ray film or LICOR/Image Studio Lite software. Prior to reblotting, polyvinylidene difluoride membranes were stripped with buffer (100 mM β-mercaptoethanol, 2% SDS and 62.5 mM Tris–HCL [pH 6.7]) at 55 °C for 30 min.

Microcapillary liquid chromatography-tandem mass spectrometry (LC–MS/MS) was performed by the Taplin Mass Spectrometry Facility (Harvard University) using methods for phosphorylation analysis. Phosphorylation sites were compared between unstimulated and stimulated cells (IL-2 or TCR stimulation) to identify inducible phosphorylation events from lower and upper bands. Briefly, excised gel bands were reduced and then alkylated prior to in-gel trypsin digestion^38^. Washed, and dehydrated gel pieces were rehydrated with 50 mM ammonium bicarbonate solution containing 12.5 ng/µl modified sequencing-grade trypsin (Promega, Madison, WI) at 4 °C, prior to 37 °C overnight. Peptides were extracted and dried by speed-vac (1 h) and stored at 4 °C until analysis. Reconstituted samples (5–10 ul of 2.5% acetonitrile, 0.1% formic acid) were loaded onto a nano-scale reverse-phase HPLC capillary column created by packing 2.6 um C18 spherical silica bads into a fused silica capillary (100 um inner diameter × ~ 30 cm length) with a flame drawn tip^39^ via a Famos auto sampler (LC Packings). A gradient was formed and peptides were eluted with increasing concentrations of 97.5% acetonitrile, 0.1% formic acid. Eluted peptides were subjected to electrospray ionization (doubly charged) and entered into a LTQ Orbitrap Velos Pro ion-trap mass spectrometer for detection, isolation and fragmentation of each peptide. Protein identity was determined by matching peptides with the acquired fragmentation pattern using Sequest (ThermoFinnigan)^40^. Phosphorylation of serine, threonine, or tyrosine (79.9663 mass units) was included in the database searches to determine phosphopeptides and phosphorylation assignments were determined by the Ascore algorithm^41^. Further, analysis was performed by the Border Biomedical Research Center (BBRC) Core Biomolecule Analysis laboratory as follows. Proteomic data analysis was performed using the Proteome Discoverer (PD) v2.5.0.400 (Thermo Fisher Scientific), with an estimated false discovery rate (FDR) of 1%. Common contaminants such as trypsin autolysis fragments, human keratins, and protein lab standards, were included as well as in house contaminants which may be found in the cRAP contaminant database. Crk-like protein, P46109, was downloaded from UniProtKB; http://www.uniprot.org/ (Downloaded 2021.19.07). The following parameters were used in the PD: HCD MS/MS; fully tryptic peptides only; up to 1 missed cleavage; parent- ion mass tolerance of 10 ppm (monoisotopic); and a liberal fragment mass tolerance of 1 Da (in Sequest HT). Cysteine carbamidomethylation was included as a fixed modification. Oxidation of methionine and deamidation of asparagine and glutamine as well as phosphorylation of serine sites were allowed as variable modifications. Spectra and Peptide Spectral Matching Identifications of potential phosphorylation sites (CrkL S114) were included with a cut off value of > 2 XCorr values to validate phosphorylated peptide fragments.

Anti-CrkL pS114 polyclonal containing antiserum was generated by Genscript using the human CrkL peptide sequence (MGSVpSAPNLPTAEDC). Dot blots with pS114 antibody were conducted using these same non-phosphorylated and phosphorylated peptides. Prior to Western blotting, anti-serum was pre-blocked with non-phosphopeptide (MGSVSAPNLPTAEDC) for 2 h; rotating end-over-end at room temperature, followed by dilution in blocking buffer (1% bovine serum albumin, 2% sodium azide) at 1:1000.

### [^32^P]-orthophosphate labeling and phosphoamino acid analysis

YT cells were metabolically labeled using [^32^P]-orthophosphate, as previously described^37^. Cells were lysed and immunoprecipitated with CrkL antibodies, captured by protein A sepharose beads, eluted, and separated by SDS-PAGE, then transferred to PVDF membrane and visualized using autoradiography. Proteins were visualized using Coomassie Blue stain (Bio-Rad) and autoradiography, then excised and subjected to limited hydrolysis in 6 N HCL at 100 °C for 30 min. Samples were then dried and resuspended in pH 1.90 buffer (Formic acid, acetic acid and water at a 10:100:1890 ratio) containing 1 µg of phosphoamino acid standards (pY, pS, pT). The samples were subsequently spotted onto a thin layer cellulose-acetate gel, and electrophoresis was performed in the first dimension at 1500 V for 30 min in pH 3.9 buffer (Pyridine:acetic acid:water at 10:100:1890 ratio) using the Hunter Thin Layer Electrophoresis apparatus^42^. Standards were visualized with ninhydrin, and radiolabeled samples were detected by autoradiography.

### Statistical analysis

Statistical analysis was performed by One-way ANOVA and Tukey test using Prism 8.2 software.

## Supplementary Information

Supplementary Information.
