# Supplementary material for: Phosphorylation of CrkL S114 induced by common gamma chain cytokines and T-cell receptor signal transduction
Source: Sci Rep. 2021 Aug 20;11:16951. doi: 10.1038/s41598-021-96428-y (PMC8379229; doi:10.1038/s41598-021-96428-y)
Supplement: Supplementary file 1 — Supplementary Information. [file 41598_2021_96428_MOESM1_ESM.pdf]

Supplementary information

Phosphorylation of CrkL S114 induced by common gamma chain cytokines and T-cell Receptor signal transduction

Armando Estrada III[1,2], **Alejandro C. Rodriguez**[1,2,+], **Georgialina Rodriguez**[1,2,+], **Alice H. Grant**[1,2,+], **Yoshira M. Ayala-Marin** [1,2,+], **Amy J. Arrieta**[1,+] **and Robert A. Kirken**[1,2,*]

[1]Department of Biological Sciences, The University of Texas at El Paso, El Paso, TX 79968, U.S.A.
[2]Border Biomedical Research Center, The University of Texas at El Paso, El Paso, TX 79968, U.S.A.
[*]Corresponding author, Robert A. Kirken, rkirken@utep.edu
[+]These authors contributed equally to this work

a

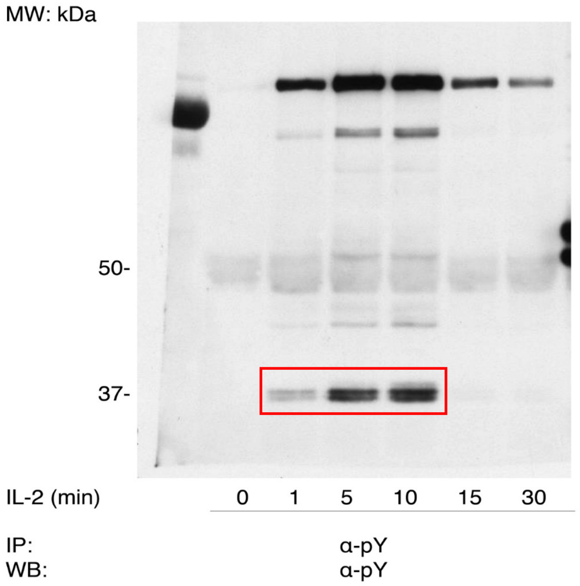

b

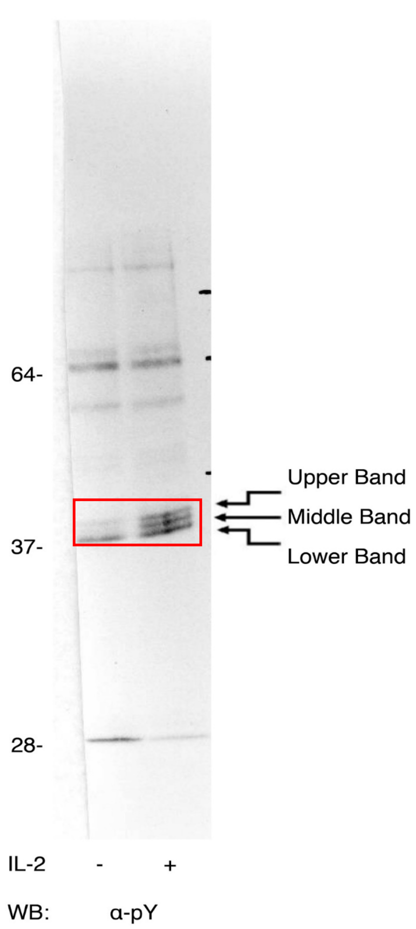

Supplementary Figure. S1. Full-length Western blots. (**a**) Full-length Western blot image for Fig. 1a. (**b**) Full-length Western blot image for Fig. 1b

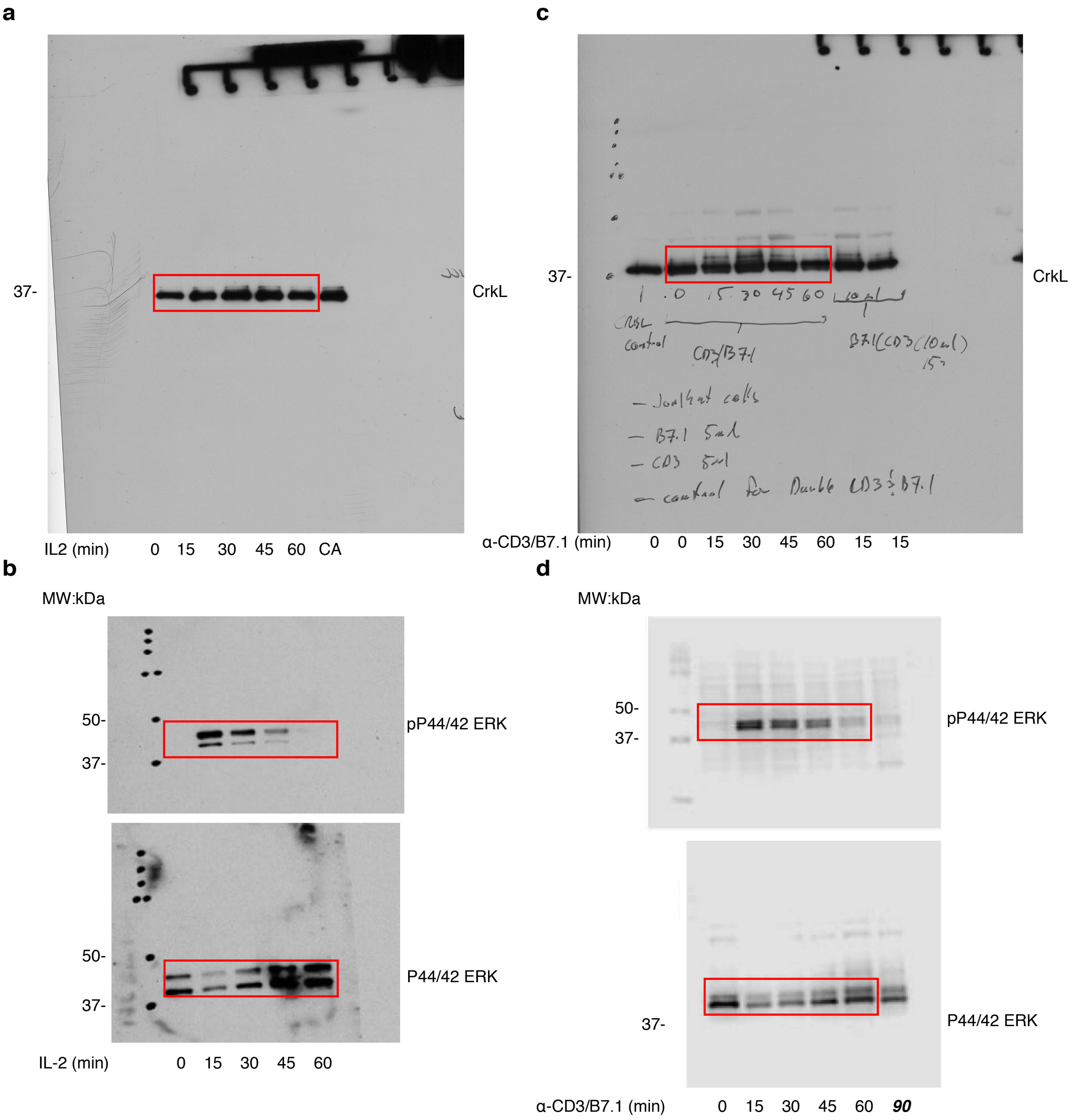

Supplementary Figure. S2. Full-length Western blots. (**a**) Full-length Western blot image for Fig. 2a upper panel. Last lane includes a sample treated with Calyculin A (CA) that was not shown in the manuscript. (**b**) Full-length Western blot image for Fig. 2a middle and lower panel. (**c**) Full-length Western blot image for Fig. 2c upper panel. First lane includes a CrkL loading control and last two lanes include samples treated with a higher concentration of αCD3/B7.1 that were not shown in the manuscript. (**d**) Full-length Western blot image for Fig. 2c middle and lower panel. Western blot images for ERK were generated using LICOR/Image Studio Lite software.

a

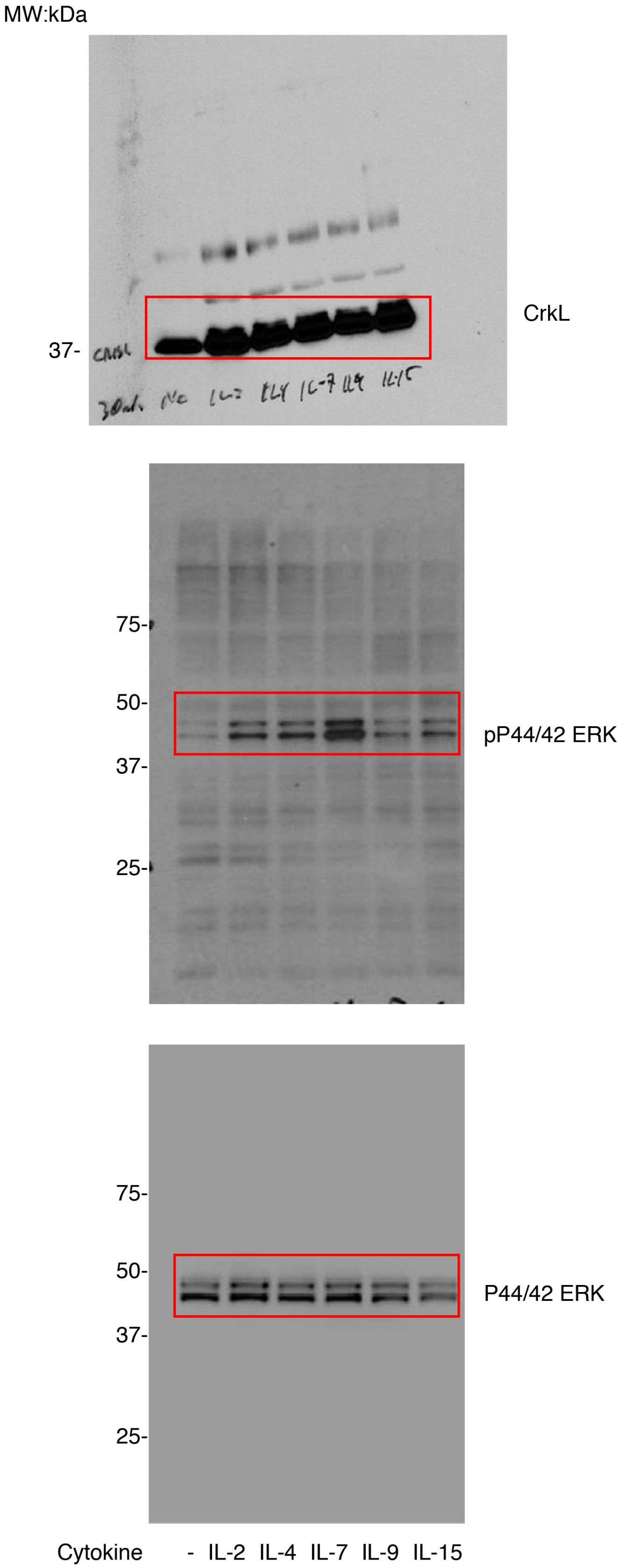

Supplementary Figure. S3. Full-length Western blots. (**a**) Full-length Western blot image for Fig. 3a. Western blot images

for ERK were generated using LICOR/Image Studio Lite software.

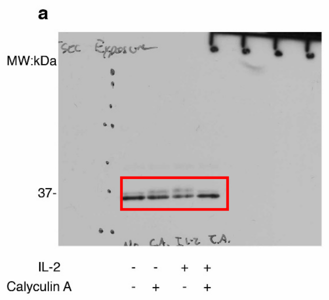

b⁺	b²⁺	b³⁺	Ion Series Seq.	y⁺	y²⁺	y³⁺	b⁺Phos	b²⁺Phos	b³⁺Phos	Modification Losses Seq.	y⁺Phos	y²⁺Phos	y³⁺Phos
164.0706	82.53894	55.36172	Y							P	2539.213	1270.11	847.076
261.1234	131.0653	87.71264	P	2637.19	1319.099	879.7349				S	2442.161	1221.584	814.725
348.1554	174.5813	116.7233	S	2540.137	1270.572	847.384				P	2355.129	1178.068	785.7144
445.2082	223.1077	149.0742	P	2453.105	1227.056	818.3733				P	2258.076	1129.542	753.3634
542.2609	271.6341	181.4252	P	2356.053	1178.53	786.0224				M-Oxidation	2161.023	1081.015	721.0125
689.2963	345.1518	230.437	M-Oxidation	2259	1130.004	753.6715				G	2013.988	1007.497	672.0007
746.3178	373.6625	249.4441	G	2111.965	1056.486	704.6597				S	1956.966	978.9867	652.9936
833.3498	417.1786	278.4548	S	2054.943	1027.975	685.6525				V	1869.934	935.4707	623.9829
932.4182	466.7128	311.4776	V	1967.911	984.4591	656.6419	1001.44	501.2235	334.4848	S-Phospho	1770.866	885.9365	590.9601
1099.417	550.2119	367.1437	S-Phospho	1868.843	934.9249	623.6191	1072.477	536.742	358.1638	A			
1170.454	585.7305	390.8228	A	1701.844	851.4258	567.9529	1169.53	585.2684	390.5147	P			
1267.506	634.2569	423.1737	P	1630.807	815.9072	544.2739	1283.573	642.2899	428.529	N			
1381.549	691.2783	461.188	N	1533.754	767.3808	511.923	1396.657	698.8319	466.2237	L			
1494.633	747.8204	498.8827	L	1419.711	710.3594	473.9087	1493.709	747.3583	498.5746	P			
1591.686	796.3468	531.2336	P	1306.627	653.8173	436.214	1594.757	797.8821	532.2572	T			
1692.734	846.8706	564.9162	T	1209.575	605.2909	403.8631	1665.794	833.4007	555.9362	A			
1763.771	882.3892	588.5952	A	1108.527	554.7671	370.1805	1794.837	897.922	598.9504	E			
1892.814	946.9104	631.6094	E	1037.49	519.2486	346.5015	1909.864	955.4355	637.2927	D			
2007.841	1004.424	669.9517	D	908.4472	454.7273	303.4873	2023.907	1012.457	675.3071	N			
2121.883	1061.445	707.966	N	793.4203	397.2138	265.1449	2136.991	1068.999	713.0017	L			
2234.968	1117.987	745.6607	L	679.3774	340.1923	227.1306	2266.033	1133.52	756.0159	E			
2364.01	1182.509	788.6749	E	566.2933	283.6503	189.436	2429.097	1215.052	810.3704	Y			
2527.073	1264.04	843.0293	Y	437.2507	219.129	146.4218	2528.165	1264.586	843.3932	V			
2626.142	1313.575	876.0521	V	274.1874	137.5973	92.06731				R			
			R	175.119	88.06311	59.0445							

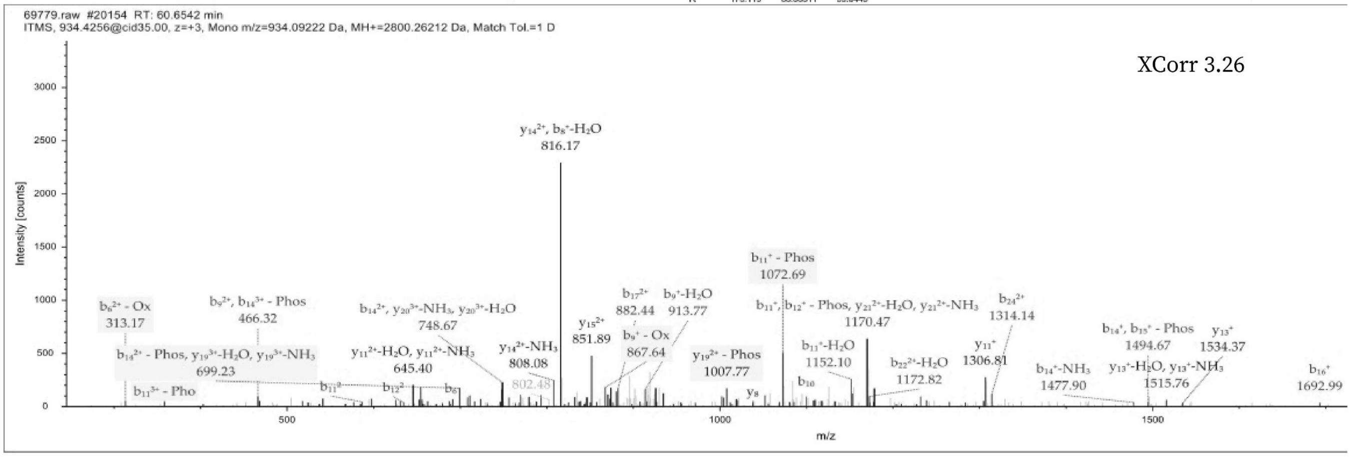

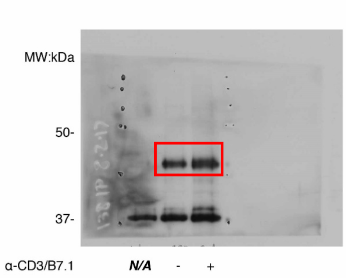

b⁺	b²⁺	b³⁺	Ion Series Seq.	y⁺	y²⁺	y³⁺	b⁺Phos	b²⁺Phos	b³⁺Phos	Modification Losses Seq.	y⁺Phos	y²⁺Phos	y³⁺Phos
164.0706	82.53894	55.36172	Y							P	2539.21333	1270.1103	847.07596
261.1234	131.0653	87.71264	P	2637.19	1319.099	879.7349				S	2442.16057	1221.58392	814.72504
348.1554	174.5813	116.7233	S	2540.137	1270.572	847.384				P	2355.12854	1178.06791	785.71436
445.2082	223.1077	149.0742	P	2453.105	1227.056	818.3733				P	2258.07577	1129.54152	753.36344
542.2609	271.6341	181.4252	P	2356.053	1178.53	786.0224				M-Oxidation	2161.02301	1081.01514	721.01252
689.2963	345.1518	230.437	M-Oxidation	2259	1130.004	753.6715				G	2013.98761	1007.49744	672.00072
746.3178	373.6625	249.4441	G	2111.965	1056.486	704.6597				S	1956.96615	978.98671	652.99357
833.3498	417.1786	278.4548	S	2054.943	1027.975	685.6525				V	1869.93412	935.4707	623.98289
932.4182	466.7128	311.4776	V	1967.911	984.4591	656.6419	1001.43969	501.22349	334.48475	S-Phospho	1770.8657	885.93649	590.96009
1099.417	550.2119	367.1437	S-Phospho	1868.843	934.9249	623.6191	1072.47681	536.74204	358.16379	A			
1170.454	585.7305	390.8228	A	1701.844	851.4258	567.9529	1169.52957	585.26842	390.51471	P			
1267.506	634.2569	423.1737	P	1630.807	815.9072	544.2739	1283.5725	642.28989	428.52902	N			
1381.549	691.2783	461.188	N	1533.754	767.3808	511.923	1396.65656	698.83192	466.22371	L			
1494.633	747.8204	498.8827	L	1419.711	710.3594	473.9087	1493.70933	747.3583	498.57463	P			
1591.686	796.3468	531.2336	P	1306.627	653.8173	436.214	1594.75701	797.88214	532.25719	T			
1692.734	846.8706	564.9162	T	1209.575	605.2909	403.8631	1665.79412	833.4007	555.93622	A			
1763.771	882.3892	588.5952	A	1108.527	554.7671	370.1805	1794.83671	897.92199	598.95042	E			
1892.814	946.9104	631.6094	E	1037.49	519.2486	346.5015	1909.86366	955.43547	637.29274	D			
2007.841	1004.424	669.9517	D	908.4472	454.7273	303.4873	2023.90658	1012.45693	675.30705	N			
2121.883	1061.445	707.966	N	793.4203	397.2138	265.1449	2136.99065	1068.99896	713.00173	L			
2234.968	1117.987	745.6607	L	679.3774	340.1923	227.1306	2266.03324	1133.52026	756.01593	E			
2364.01	1182.509	788.6749	E	566.2933	283.6503	189.436	2429.09657	1215.05192	810.37037	Y			
2527.073	1264.04	843.0293	Y	437.2507	219.129	146.4218	2528.16498	1264.58613	843.39318	V			
2626.142	1313.575	876.0521	V	274.1874	137.5973	92.06731				R			
			R	175.119	88.06311	59.0445							

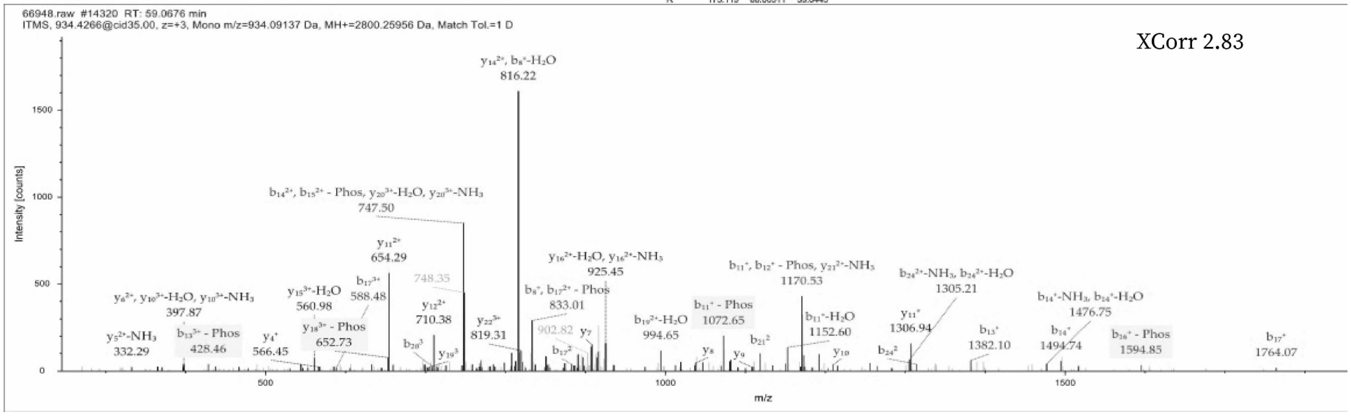

Supplementary Figure. S4. Full-length Western blots and Mass Spectrometry Data. (**a**) Full-length Western blot image for

Fig. 4a and corresponding Mass Spectrum is shown with S114 post-translationally modified in response to IL-2 as indicated by Phos and light shading. (**b**) Full-length Western blot image for Fig. 4b. Not Applicable (N/A) lane was used to test an alternate monoclonal CrkL antibody that was not included in the manuscript. Corresponding Mass Spectrum is shown with S114 post-translationally modified in response to αCD3/B7.1 stimulation as indicated by Phos and light shading.

Supplementary Table 1. XCorr and Ascore values for S114 phospho-peptides.

CrkL Site	Stimulation	Modification	XCorr	Ascore
S114	IL2	Phosphorylation	3.26	11
S114	TCR mimicry	Phosphorylation	2.83	17.3

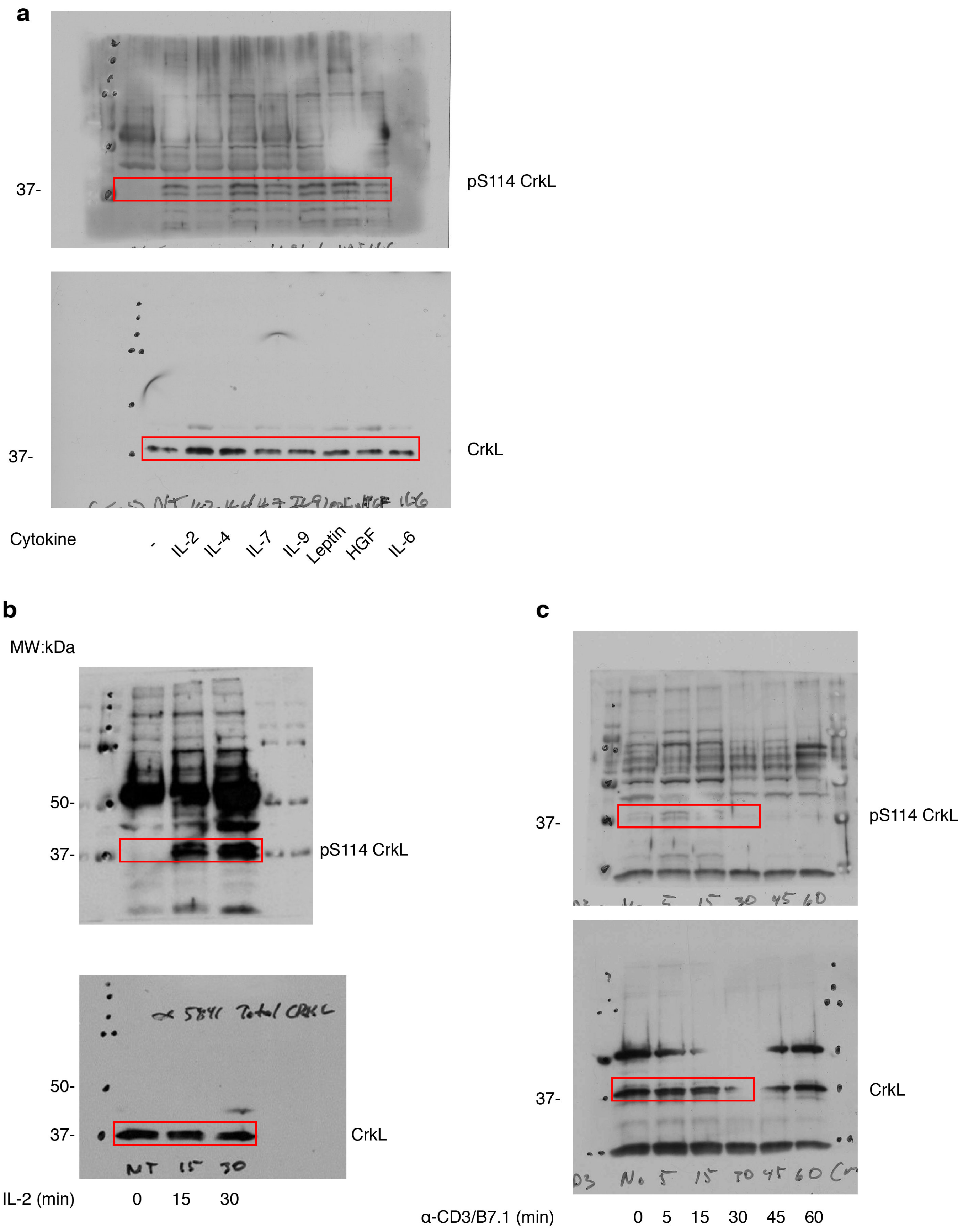

a
37-
pS114 CrkL
37-
CrkL
Cytokine
-
IL-2
IL-4
IL-7
IL-9
Leptin
HGF
IL-6
b
MW:kDa
50-
37-
pS114 CrkL
50-
37-
CrkL
IL-2 (min)
0
15
30
c
37-
pS114 CrkL
37-
CrkL
α-CD3/B7.1 (min)
0
5
15
30
45
60

Supplementary Figure. S5. Full-length Western blots. (**a**) Full-length Western blot images for Fig. 5b. (**b**) Full-length Western blot images for Fig. 5c. (**c**) Full-length Western blot images for Fig. 5d.

a

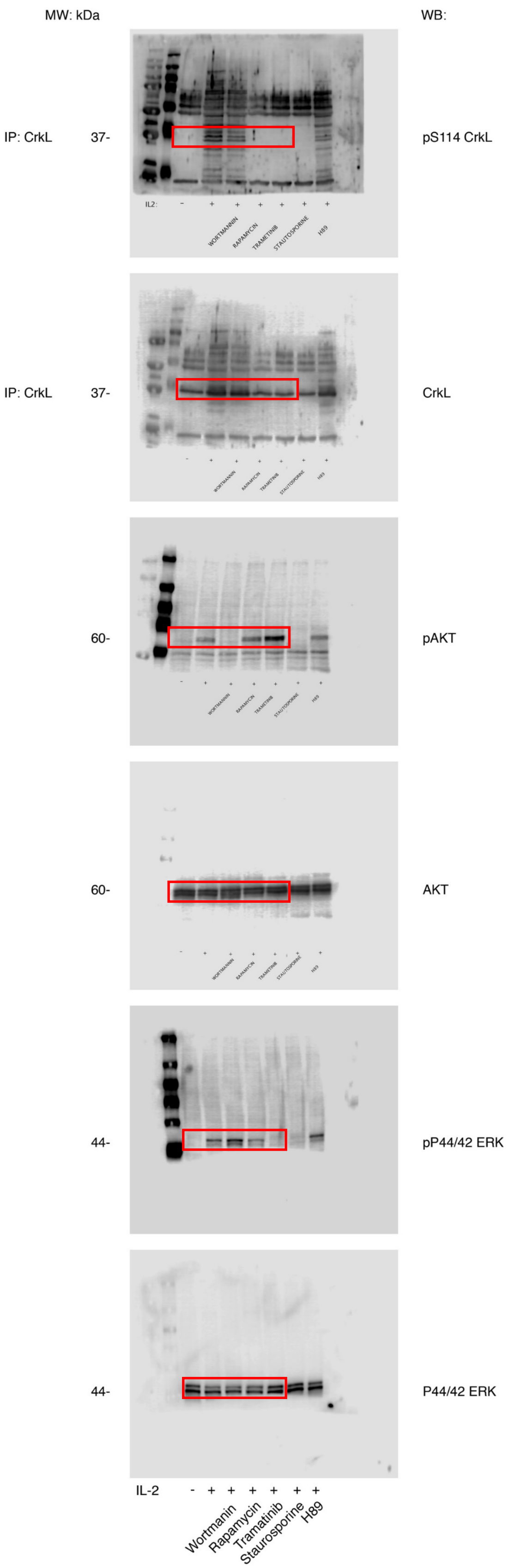

Supplementary Figure. S6. Full-length Western blots. (**a**) Full-length Western blot image for Fig. 6a. The last two lanes

include samples treated with non-specific kinase inhibitor staurosporine (100 nM, PKC inhibitor, Selleck Chemicals), and H89 (100 nM, PKA inhibitor) and were not included in the manuscript. Images were generated using LICOR/Image Studio Lite software.
